# Aquaporins in sepsis- an update

**DOI:** 10.3389/fimmu.2024.1495206

**Published:** 2024-10-31

**Authors:** Katharina Rump, Michael Adamzik

**Affiliations:** Klinik für Anästhesiologie Intensivmedizin und Schmerztherapie Universitätsklinikum Knappschaftskrankenhaus Bochum, University Clinic of Ruhr University Bochum, Bochum, Germany

**Keywords:** aquaporin (AQP), AQP9 aquaporin-9, drug target, sepsis, pathophysiology sepsis, AQP5 aquaporin 5, AQP3, immune metabolism

## Abstract

Aquaporins (AQPs), a family of membrane proteins that facilitate the transport of water and small solutes, have garnered increasing attention for their role in sepsis, not only in fluid balance but also in immune modulation and metabolic regulation. Sepsis, characterized by an excessive and dysregulated immune response to infection, leads to widespread organ dysfunction and significant mortality. This review focuses on the emerging roles of aquaporins in immune metabolism and their potential as therapeutic targets in sepsis, with particular attention to the modulation of inflammatory responses and organ protection. Additionally, it explores the diverse roles of aquaporins across various organ systems, highlighting their contributions to renal function, pulmonary gas exchange, cardiac protection, and gastrointestinal barrier integrity in the context of sepsis. Recent studies suggest that AQPs, particularly aquaglyceroporins like AQP3, AQP7, AQP9, and AQP10, play pivotal roles in immune cell metabolism and offer new therapeutic avenues for sepsis treatment. In the context of sepsis, immune cells undergo metabolic shifts to meet the heightened energy demands of the inflammatory response. A key adaptation is the shift from oxidative phosphorylation (OXPHOS) to aerobic glycolysis, where pyruvate is converted to lactate, enabling faster ATP production. AQPs, particularly aquaglyceroporins, may facilitate this process by transporting glycerol, a substrate that fuels glycolysis. AQP3, for example, enhances glucose metabolism by transporting glycerol and complementing glucose uptake via GLUT1, while also regulating O-GlcNAcylation, a post-translational modification that boosts glycolytic flux. AQP7 could further contributes to immune cell energy production by influencing lipid metabolism and promoting glycolysis through p38 signaling. These mechanisms could be crucial for maintaining the energy supply needed for an effective immune response during sepsis. Beyond metabolism, AQPs also regulate key immune functions. AQP9, highly expressed in septic patients, is essential for neutrophil migration and activation, both of which are critical for controlling infection. AQP3, on the other hand, modulates inflammation through the Toll-like receptor 4 (TLR4) pathway, while AQP1 plays a role in immune responses by activating the PI3K pathway, promoting macrophage polarization, and protecting against lipopolysaccharide (LPS)-induced acute kidney injury (AKI). These insights into the immunoregulatory roles of AQPs suggest their potential as therapeutic targets to modulate inflammation in sepsis. Therapeutically, AQPs present promising targets for reducing organ damage and improving survival in sepsis. For instance, inhibition of AQP9 with compounds like HTS13286 or RG100204 has been shown to reduce inflammation and improve survival by modulating NF-κB signaling and decreasing oxidative stress in animal models. AQP5 inhibition with methazolamide and furosemide has demonstrated efficacy in reducing immune cell migration and lung injury, suggesting its potential in treating acute lung injury (ALI) in sepsis. Additionally, the regulation of AQP1 through non-coding RNAs (lncRNAs and miRNAs) may offer new strategies to mitigate organ damage and inflammatory responses. Moreover, AQPs have emerged as potential biomarkers for sepsis progression and outcomes. Altered expression of AQPs, such as AQP1, AQP3, and AQP5, correlates with sepsis severity, and polymorphisms in AQP5 have been linked to better survival rates and improved outcomes in sepsis-related acute respiratory distress syndrome (ARDS). This suggests that AQP expression could be used to stratify patients and tailor treatments based on individual AQP profiles. In conclusion, AQPs play a multifaceted role in the pathophysiology of sepsis, extending beyond fluid balance to crucial involvement in immune metabolism and inflammation. Targeting AQPs offers novel therapeutic strategies to mitigate sepsis-induced organ damage and improve patient survival. Continued research into the metabolic and immune functions of AQPs will be essential for developing targeted therapies that can be translated into clinical practice.

## 1 Background

Sepsis represents a prevalent complication in Intensive Care Units across Germany and the United States (1), with persistently high mortality due to its complex immunological nature. In the United States septic conditions accounts for more than $22 billion (11.2%) of total US hospital costs in 2017 (2). Incidence and mortality of sepsis differ among the regions worldwide. Incidence reaches from 158/100 000 population in 2015 in Germany (3) to 780/100000 population in Sweden (4) For patients with clearly documented sepsis (including severe sepsis), the mortality rates from 2010 to 2015 fell from 26.6% to 23.5%, while for those with severe sepsis alone, the rates decreased from 47.8% to 41.7%. in Germany (5). These figures are comparable to the rates in England (6) but significantly higher than those in the USA (15%) and Australia (18.4%) (7, 8).The absence of predictive biomarkers specific to this syndrome prevents tailored individual therapies based on patients’ immune status. Aquaporins (AQPs) are potential biomarkers due to their significant roles in inflammation, particularly in sepsis, as evidenced by experimental and association studies (9–11). AQPs are emerging as promising candidates in sepsis research due to their significant roles in inflammation and immune responses (12). Experimental and association studies indicate that AQPs are not merely transport proteins; their dysregulation is observed in immune and epithelial cells when exposed to infectious and inflammatory stimuli (13). Recent findings have firmly established the involvement of AQPs in inflammatory processes, particularly since several AQP isoforms are expressed in both innate and adaptive immune cells (14). They play crucial roles in phagocytic functions and specific immune processes such as cell activation and migration (15, 16).

The recognition of AQPs in inflammation enhances our understanding of the complex mechanisms governing host-pathogen interactions. As such, AQPs represent potential therapeutic targets for modulating edema, cell migration, and the release of inflammatory cytokines and mediators (17, 18).

Aquaporins are a family of membrane proteins that facilitate the transport of water across biological membranes. They are integral to maintaining water balance in cells and tissues. As described, Aquaporins are essential for water homeostasis in all organisms (19). These proteins are known for their remarkable ability to transport water selectively and efficiently (20). Aquaporins play critical roles in various physiological processes, including kidney water conservation, brain water balance, and secretion of cerebrospinal fluid (19).

AQPs comprise a group of 13 membrane proteins crucial for regulating cellular water, salt fluxes, and the transport of small solutes like glycerol, urea, and carbon dioxide (17). Water-selective AQPs play roles in transepithelial fluid transport, cell migration, brain edema, and neuroexcitation (17), while aquaglyceroporins are involved in cell proliferation, adipocyte metabolism, and epidermal water retention. **Table 1** illustrates the various families of aquaporins. The objective of this study is to provide an update on the potential contributions of aquaporins (AQPs) to the pathomechanisms of sepsis, based on the current literature findings (21).

**Table 1:** the different AQP subfamilies and their permeability according to (22).

Subfamily	Aquaporin	permeability
Classical AQPs (water-channels)	AQP0	H_2_O, H_2_O_2_
	AQP1	H_2_O, CO_2_, NH_3_
	AQP2	H_2_O
	AQP4	H_2_O
	AQP5	H_2_O, CO_2_
	AQP6	H_2_O (pH dependent), urea, glycerol, nitrate
Aquaglyceroporins	AQP3	H_2_O, glycerol, urea, H_2_O_2_
	AQP7	H_2_O, glycerol, urea, ammonia, arsenite, NH_3_
	AQP9	Glycerol, NH_3_, urea, lactate, purine, pyrimdine, H_2_O_2_
	AQP10	Glycerol, urea, H_2_O
Superaquaporins	AQP11	H_2_O, H_2_O_2_, glycerol
	AQP12	Function less well understood, likely H_2_O
aquaammoniaporin	AQP8	H_2_O, ammonia, glycerol, H_2_O_2_

classical AQPs (green), Aquaglyceroporins (blue), superquaporins (orange) and aquaammoniaporins (red) are displayed.

In conclusion, aquaporins exert a significant influence on the pathophysiology of sepsis, affecting fluid balance, organ function and the inflammatory response. Further research is required to fully explore the potential of targeting aquaporins as a therapeutic strategy. The following paragraph will delineate the role of aquaporins in various organ systems.

## 2 The significance of aquaporins in various organ systems during sepsis

### 2.1 Aquaporins in whole blood of septic patients

**Figure 1:**
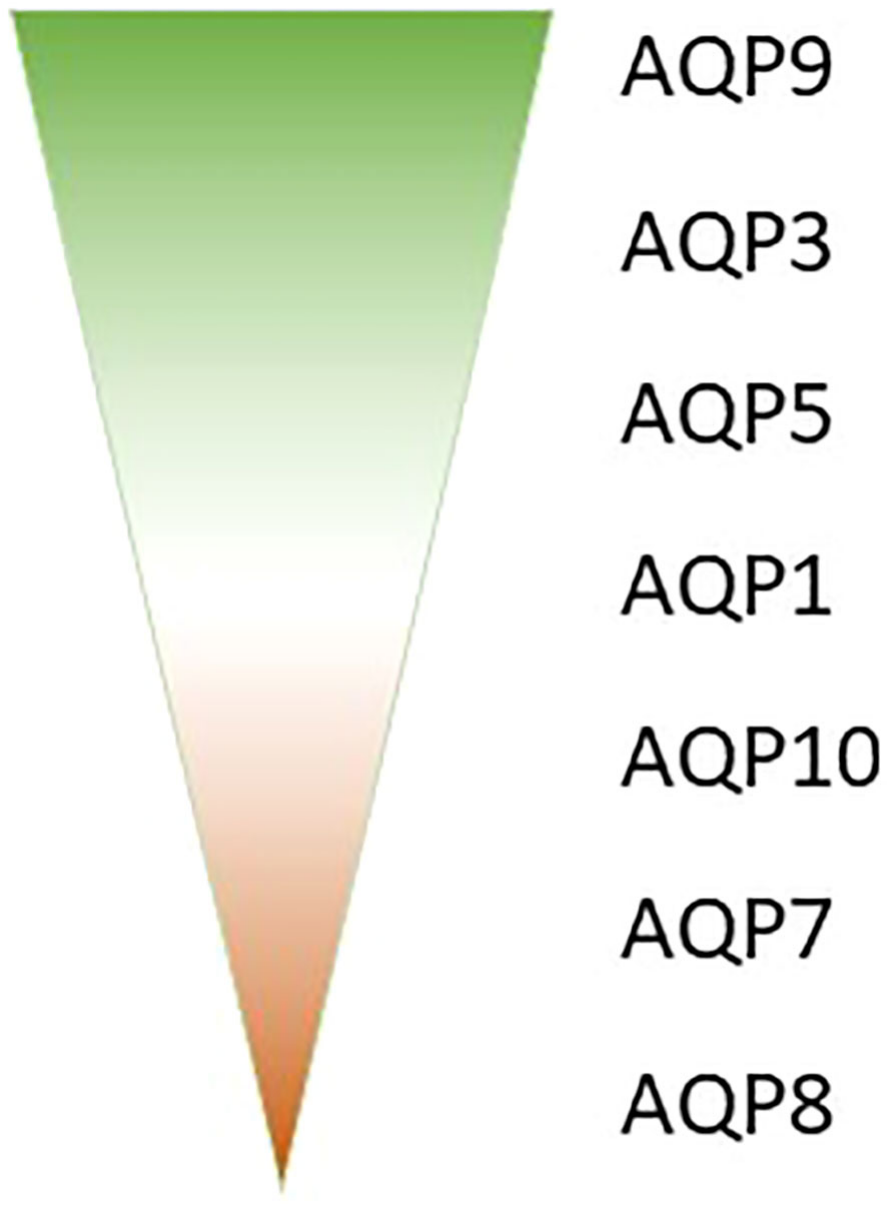
amount of AQP expression in whole blood from high (AQP9) to low amount (AQP8).

Aquaporins (AQPs) play a crucial role in the immune response, with various isoforms implicated in different immune cell types and inflammatory conditions. The AQP expression analysis in whole blood samples from septic patients might reveal valuable AQP biomarkers in sepsis. Our research analyzed those samples of septic patients and revealed that AQP9 is the most abundantly expressed aquaporin in blood, followed by AQP3, AQP5, and AQP1 (**Figure 1**). In contrast AQP10, AQP7 and AQP8 very only expressed in a small amount in whole blood (**Figure 1**) (23). The different expression in whole blood could be related to different amount of blood cells, as e.g. AQP9 is mostly expressed in high abundant neutrophils (24) and AQP3 in the second most present T-cells (25, 26). Furthermore, the expression of these aquaporins was observed to undergo varying changes between day 1 and day 8 of sepsis (23).

**Figure 2:**
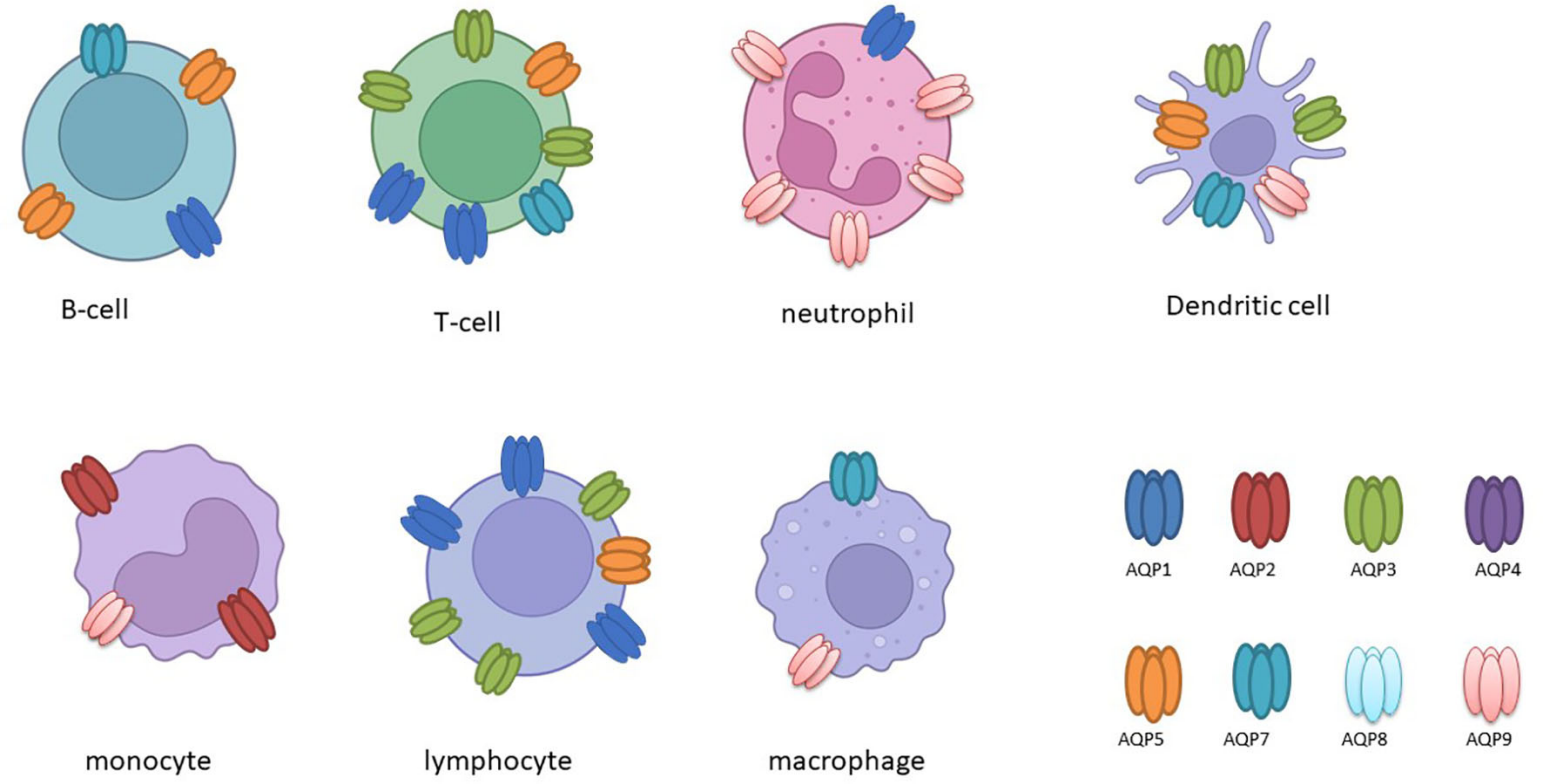
The following schematic overview, created with BioRender, depicts the expression of aquaporin (AQP) in various immune cells.

The expression of aquaporins is subject to differential regulation in immune cells (as illustrated in **Table 2** and **Figure 2**), and this is further influenced by the presence of inflammatory stimuli (10, 27).

**Table 2:** Distribution of aquaporins different immune cells (HPA: humanproteinatlas.org).

AQP	Immune cell	reference
AQP0	Not detected	HPA
AQP1	Lymphocytes (memory T-cells), leucocytes, activated B and T-lymphocytes	HPA (13, 28, 29)
AQP2	monocytes	HPA
AQP3	Dendritic cells, lymphocytes especially T-cells, (T-reg MAIT T-cell Memory CD4 T-cell Naive CD4 T-cell Memory CD8 T-cell Naive CD8 T-cell) activated B and T-lymphocytes	HPA (13, 28)
AQP4	Not detected	HPA
AQP5	Not detected to lymphocytes, dendritic cells activated B and T-lymphocytes	HPA (13, 28)
AQP6	Very low in all immune cells	HPA
AQP7	Very low T-cells and B-cells, dendritic cells, macrophages	HPA (28, 29)
AQP7B	Very low Basophil Plasmablast	HPA
AQP8	Very low T-cells	HPA
AQP9	Very high neutrophils, medium monocytes, leucocytes, macrophages, dendritic cells, HL-60 cells	HPA (28, 29)
AQP10	Very low PBMCs	HPA
AQP11	Low in all immune cells	HPA
AQP12A	Not detected	HPA
AQP12B	Not detected	HPA

It has been demonstrated that activated B and T lymphocytes express AQP1, AQP3, and AQP5, whereas immature dendritic cells (DCs) predominantly express AQP3 and AQP5. This expression correlates with the activation and proliferation of these cells (13). In leukocytes, the expression of AQP1 and AQP9 is increased following activation or stimulation with lipopolysaccharide (LPS), a component of bacterial cell walls. Furthermore, in cases of ICU-acquired sepsis and SIRS, there is a notable alteration in the expression of AQP1 and AQP9 in leukocytes, which may play a role in cellular responses and plasma membrane dynamics under inflammatory conditions (30–32). Furthermore, studies have demonstrated that LPS administration results in increased AQP1 expression and decreased AQP5 mRNA levels in THP-1 cells, underscoring the existence of isoform-specific responses in the context of inflammation (27). It is noteworthy that elevated AQP5 mRNA expression has been linked to unfavorable outcomes in sepsis patients, underscoring its potential as a prognostic marker (33).

Similarly, stimulation with lipopolysaccharide (LPS) has been observed to upregulate AQP3 in monocytic THP-1 cells, which are a model for studying macrophage activation and inflammation. Inhibition or silencing of AQP3 in these cells has been demonstrated to attenuate LPS-induced priming and reduce the production of inflammatory cytokines such as IL-6, pro-IL-1β, and TNF-α, indicating its involvement in Toll-like receptor 4 (TLR4) signalling (34).

In primary human macrophages and neutrophils, AQP9 is highly expressed and increases at both the transcript and protein levels following LPS stimulation, indicating its role in innate immune response modulation (34). AQP9, in particular, demonstrates augmented expression in activated polymorphonuclear leukocytes during systemic inflammatory response syndrome (SIRS) and infective endocarditis (32, 35). In dendritic cells (DCs), AQP9 is markedly expressed and markedly upregulated by LPS (**Figure 2**). However, the blockade of AQP9 in mice with induced colitis only partially reduces DC inflammatory responses (36). AQP9 plays a regulatory role in neutrophil migration and is associated with sepsis survival. AQP9 regulates neutrophil migration and affects sepsis survival In leukocytes, AQP9 is located at the cell edge and is thought to be involved in motility, lamellipodium extension and stabilisation, and changes in cell volume that facilitate migration towards chemoattractants (37). A further study demonstrated that the AQP9-G-quadruplex forming sequence containing long non-coding RNA (lncRNA) axis plays a pivotal role in the exacerbation of sepsis by promoting neutrophil activation and neutrophil extracellular trap (NET) release (38). It can therefore be concluded that aquaporins have specific functions in immune cells (**Figure 2**).

### 2.2 Aquaporins in sepsis-associated encephalopathy

Aquaporins (AQPs) play an important role in several aspects of sepsis-associated encephalopathy (SAE), a devastating complication of sepsis characterised by vasogenic cerebral oedema and cognitive impairment. In the context of SAE, AQPs, in particular AQP4, have been implicated in several pathological mechanisms.

**Figure 3:**
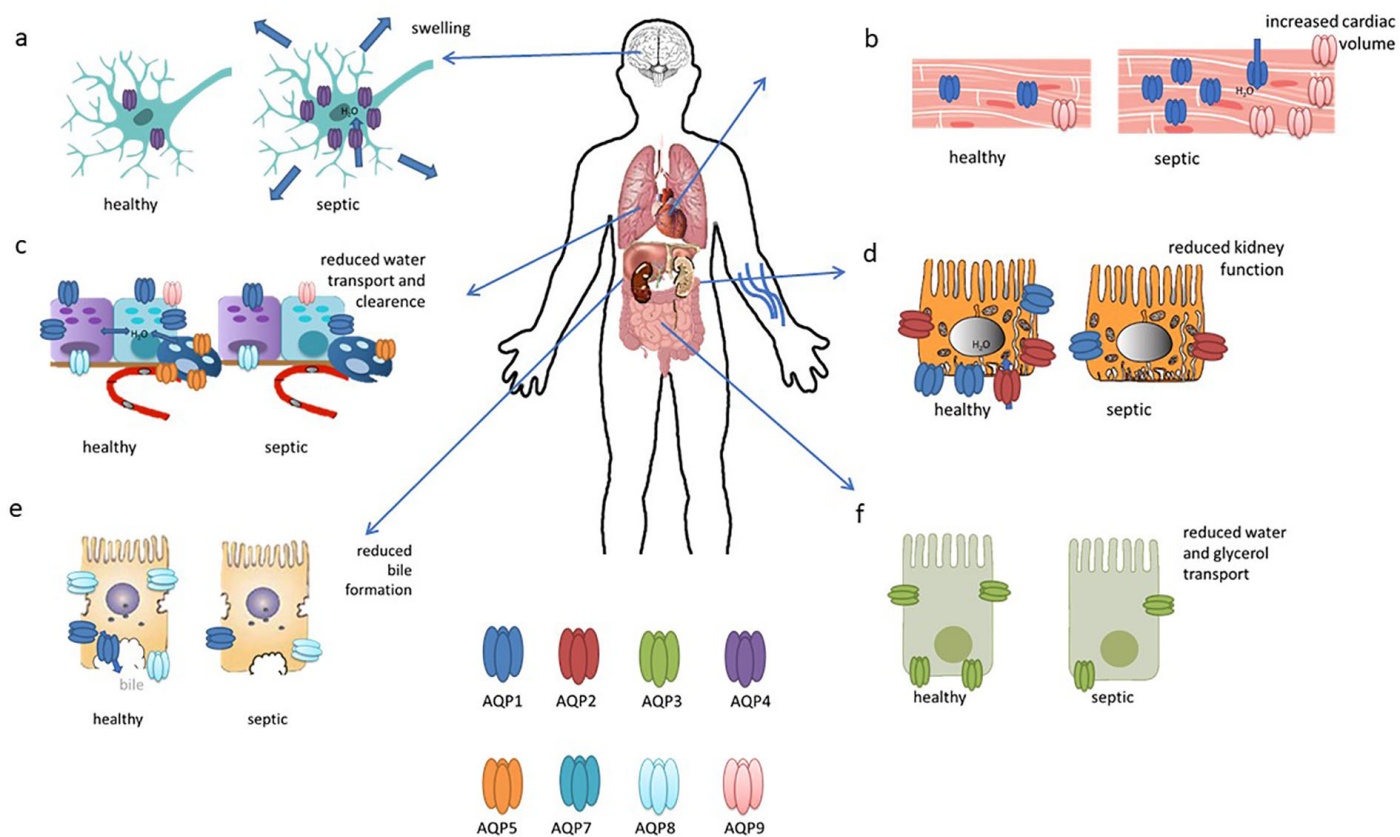
This figure illustrates the updated roles and expressions of aquaporins in differentiated organs in sepsis. **(A)** AQP4 is upregulated in the brain during sepsis; **(B)** AQP1 expression increases in cardiac cells; **(C)** AQP1, AQP8, and AQP9 are present in bronchiolar epithelial cells, while AQP5 is in alveolar epithelial cells, with all their expressions reduced in sepsis; **(D)** AQP2 is localized to the apical and subapical regions of collecting duct principal cells, with reduced expression in sepsis; **(E)** AQP8 expression decreases in hepatocytes during sepsis; **(F)** AQP3 seems to be decreased in intestinal cells. This figures has been modified and adapted from the following sources (90):and (21).

During septic encephalopathy, AQP4 is upregulated in response to cerebral inflammation mediated by neutrophil infiltration, exacerbating cerebral oedema (39–41) (**Figure 3A**). SAE is also associated with astrocytic inflammation involving AQP4. AQP4 is upregulated in the peripheral blood of SAE patients and in the brain tissue of a mouse model in which AQP4 deletion can reduce cognitive impairment by activating astrocytic autophagy and inhibiting neuroinflammation. In addition, AQP4 knockout seem to reduce Ca^2+^ accumulation and downregulated voltage-gated, type 8, alpha subunit channels in astrocytes, thereby inhibiting the Peroxisome proliferator-activated receptor gamma pathway and providing neuroprotection (42). This upregulation of AQP4 can be attenuated by dexamethasone, primarily through tumour necrosis factor alpha (TNF-α) regulation, although the use of corticosteroids in sepsis remains controversial and is recommended under certain conditions (43–45).

Elevated ammonia levels in SAE, due to non-hepatic hyperammonemia, contribute to increased AQP4 expression in astrocytes, leading to cognitive impairment (46). Fecal microbiota transplantation in animal models has shown promise in reducing ammonia levels and improving neurological outcomes by modulating AQP4 expression via the gut-brain axis (46). Furthermore, in sepsis-induced delirium (SID), AQP4 expression in astrocytes is elevated, with exosomes carrying AQP4 proteins potentially serving as biomarkers for SID (47).

In contrast a rat CLP-model showed that SAE led to impaired cerebral blood flow, alterations in grey and white matter structure, and changes in glial cell morphology without causing widespread blood-brain barrier breakdown, accompanied by reductions in neuronal cyclooxygenase-2 (COX-2) and aquaporin-4 (AQP4) expression in cortical regions and increased perivascular COX-2 expression (48).

In experimental models of sepsis, AQP4 deletion attenuates learning and memory impairment by reducing neuroinflammation, activating astrocytic autophagy, and downregulating proinflammatory cytokines (42). It was found that inhibiting LncRNA-5657 with shRNA reduced neuronal degeneration and inflammatory markers, including aquaporin 4, metallopeptidase-9, and TNF-alpha levels in the hippocampus, suggesting its potential protective role against septic brain injury (49). Additionally, studies investigating endotoxemia-induced encephalopathy have identified increased AQP4 expression in the hippocampus, suggesting its involvement in the pathogenesis of cognitive dysfunction (45, 50).

Overall, AQP4 appears to be a crucial mediator in the pathophysiology of sepsis-associated encephalopathy, involved in brain edema, cognitive impairment, and potentially serving as a target for therapeutic intervention in clinical settings.

### 2.3 Aquaporins in cardiac dysfunction in sepsis

Cardiac dysfunction in sepsis arises from a combination of factors including systemic inflammation, cardiodepressive mediators, endothelial and mitochondrial dysfunction, hypovolemia, microcirculatory disturbances, bacterial toxins, oxidative stress, and neurohormonal dysregulation (51). It has been found that endotoxin administration impairs cardiac function and induces the expression of gelsolin, AQP1 and iNOS, with ageing having a negative effect on gelsolin induction and cardiac performance; in aged mice, increased levels of AQP1, iNOS and phosphorylated STAT3 were associated with greater cardiac dysfunction in response to endotoxic stress (52). In addition, cardiac expression of AQP1, P53 and P21 was significantly increased in LPS-treated rats (53). In another study, reduced H19 and AQP1 expression, coupled with increased miR-874 levels, were observed in sepsis patients, a lipopolysaccharide (LPS)-treated mouse model and in cell culture. The results suggest that H19 acts as a ceRNA for AQP1 by sequestering miR-874, highlighting its potential as a therapeutic target for mitigating sepsis-induced myocardial dysfunction (54).In a bacterial endotoxin-induced mouse model, deletion of aquaporin 9 (AQP9) improved survival and reduced oxidative stress. A novel AQP9 inhibitor, RG100204, was found to attenuate cardiac dysfunction as well as renal dysfunction and hepatocellular injury in a cecal ligation and puncture (CLP) model of sepsis. RG100204 significantly reduced cardiac dysfunction even when administered 3 hours after the onset of sepsis, highlighting AQP9 as a promising drug target for the treatment of sepsis-induced cardiac dysfunction (55). In conclusion, it can be stated that AQP1 and, to a lesser extent, AQP9 appear to be of particular importance in the context of septic cardiac dysfunction (**Figure 3B**).

### 2.4 Aquaporins in lung injury

Acute lung injury (ALI), a severe complication of sepsis often progressing to acute respiratory distress syndrome (ARDS), is associated with high in-hospital mortality (56). A number of aquaporins (AQPs) are expressed in the lungs, with AQP1 and AQP5 being particularly prevalent in vascular endothelial cells (**Figure 3C**), alveolar type I cells, and bronchial epithelial cells. In contrast, AQP8 and AQP9 are expressed to a lesser extent (57).

In general, AQP1 and AQP5 are of great importance in the context of regulating fluid transport and inflammation in ALI. Their expressions and functions vary depending on the specific type and location of lung injury. These insights into the pathophysiology of ALI may inform therapeutic strategies aimed at mitigating lung injury and improving clinical outcomes (58). Furthermore, the interaction between brain-derived neurotrophic factor (BDNF) and AQP5 indicates the potential for a novel mechanism that could mitigate lung damage in septic conditions by inhibiting excessive autophagy in alveolar epithelial cells (59).

Studies in septic patients have demonstrated increased AQP3 and AQP5 expression in the alveolar septum during diffuse alveolar damage (60). Conversely, experimental sepsis induced by cecal ligation puncture (CLP) in rats has been linked to decreased Aqp5 expression in lung tissue, which can be mitigated by treatments like emodin and regulated by microRNAs miR-96 and miR-330 (61–63). Similarly, Aqp1 expression decreases following exposure to lipopolysaccharide (LPS) in rat lungs, a reduction that can be counteracted by therapies such as hydrogen-rich saline and parenteral vitamin C, known for their protective effects in sepsis-related lung injury (11, 64). Experimental ALI models utilising insults such as LPS, ventilation, hyperoxia and hydrochloric acid (HCl) have demonstrated an increase in AQP1 expression. Conversely, mechanical ventilation with high tidal volume has been observed to result in a reduction in pulmonary AQP1 levels, which can impact fluid balance and the development of lung oedema (65). Another study using a CLP-induced sepsis rat model, myocyte enhancer factor overexpression was found to alleviate acute lung injury by up-regulating AQP1 expression. This effect suggests AQP1 modulation as a potential therapeutic strategy for sepsis-induced ALI (66). In addition, it was demonstrated that AQP1 expression is decreased in HUVEC cells, stimulated with the inflammatory factor Tumor Necrosis Factor Receptor Superfamily, Member 11b (TNFRSF11B) (67).

AQP5, highly expressed in alveolar epithelial cells, is significantly impaired after prolonged exposure to hyperoxia, highlighting its role in maintaining water movement and preventing pulmonary edema (68).

In models of inflammatory pancreatitis, there is a reduction in the expression of Aqp1 and Aqp5 in the lung, whereas Aqp8 and Aqp9 remain unaffected. The traditional Chinese medicine Dai-Huang-Fu-Zi-Tang has been demonstrated to be effective in upregulating Aqp1 and Aqp5 and in attenuating inflammation in these scenarios (69).

Further substances have been demonstrated to modulate AQP expression in lung tissue. Emodin treatment has been shown to improve sepsis-induced lung pathology by upregulating AQP and tight junction expression, reducing inflammatory cytokines, and inhibiting pulmonary apoptosis. These findings suggest that emodin may have therapeutic potential in the treatment of sepsis-induced ALI (70). The traditional Chinese formula Da-Cheng-Qi decoction has been demonstrated to suppress the TLR4/NF-κB signalling pathway, increase AQP1 and AQP5 protein expression, and inhibit inflammatory cytokine production. These effects may contribute to the alleviation of inflammatory reactions in ALI (71). Dexamethasone pretreatment at various concentrations has been demonstrated to attenuate lipopolysaccharide (LPS)-induced suppression of cell proliferation, thereby reducing the LPS-induced reduction of aquaporin 5 (AQP5) expression and apoptosis in neonatal type II alveolar epithelial cells (72). anshinol treatment in a rat sepsis model has been demonstrated to significantly increase AQP5 mRNA expression and reduce inflammatory cytokines IL-6 and TNF-α. This suggests a protective effect on lung tissue by upregulating AQP5 through the inhibition of inflammatory pathways (73). Another study explored the impact of miR-34b-5p on sepsis-induced injury in human renal tubular epithelial cells, revealing that elevated miR-34b-5p levels in septic acute kidney injury (AKI) patients correlated with inflammation and apoptosis through downregulation of AQP2, a direct target of miR-34b-5p, exacerbating injury when overexpressed and mitigated by inhibiting miR-34b-5p or enhancing AQP2 expression Another study investigated the influence of miR-34b-5p on sepsis-induced injury in human renal tubular epithelial cells. The findings indicated that elevated miR-34b-5p levels in septic acute kidney injury (AKI) patients were associated with inflammation and apoptosis through the downregulation of AQP2, a direct target of miR-34b-5p. This resulted in an exacerbation of injury when miR-34b-5p was overexpressed and a mitigation of injury when miR-34b-5p was inhibited or AQP2 expression was enhanced (74). Furthermore, the antioxidant Ss-31 was observed to reduce AQP3 expression and ROS levels, thereby improving vascular permeability and enhancing the survival of rats with sepsis. These findings indicate that modulation of AQP3 and inhibition of ROS by Ss-31 may represent promising strategies for the treatment of sepsis-induced pulmonary complications (75). In conclusion, the regulation and expression of aquaporins, particularly AQP1 and AQP5, play a critical role in fluid transport and inflammatory responses in acute lung injury (ALI). Variations in AQP expression depending on the type and location of lung injury provide valuable insights into potential therapeutic strategies for mitigating sepsis-induced lung damage. Further research on modulating AQP expression could lead to improved clinical outcomes in patients with ALI and sepsis.

### 2.5 Aquaporins in acute kidney injury

Approximately 50% of sepsis patients develop acute kidney injury (AKI), which is associated with high mortality rates (76). AQP1 is highly expressed in the kidney and facilitates water reabsorption in the proximal tubules, the thin descending limb of Henle, and the descending vasa recta. In contrast, AQP2, AQP3, and AQP4 are localised to the principal cells of the connecting tubules and collecting ducts, which are crucial for maintaining body water homeostasis and urine concentration (77). In AKI mainly AQP1 and AQP2 seems to be involved (**Figure 3D**). The expression of AQP1 is markedly elevated in renal tissue and heart tissue of rats subjected to LPS-induced AKI, but exhibits a reduction in the lung and small intestine. This suggests that AQP1 may serve as a promising novel diagnostic biomarker for septic AKI (53). Additionally, miR-144-3p upregulation was linked to the downregulation of aquaporin-1 (AQP1), which may impact renal function during systemic inflammation induced by lipopolysaccharide (LPS) (78). AQP1 plays a role in the protection against LPS-induced acute kidney injury (AKI) by promoting M2 macrophage polarization, which involves PI3K activation. AQP1 thus modulates immune responses and indicates PI3K as a pivotal pathway in AQP1-mediated macrophage polarization during sepsis-induced AKI (79).

This conclusion is supported by other analyses using an LPS-induced HK-2 cell model of septic acute kidney injury, which demonstrated that AQP1 plays a cytoprotective role. The overexpression of AQP1 in HK-2 cells resulted in the attenuation of the LPS-induced reduction in cell viability, increase in apoptosis, and upregulation of proinflammatory cytokines and chemokines. This was achieved by the inhibition of the p38, p53 and ERK1/2 pathways, which suggests AQP1 as a potential therapeutic target for sepsis-induced acute kidney injury (80, 81).

Further AKI is associated with downregulated Aqp2 expression through the NF-κB pathway in a CLP mouse model. Pretreatment with a continuous erythropoietin receptor activator (CERA) or α-lipoic acid has been demonstrated to preserve Aqp2 expression and protect against sepsis-induced AKI. Conversely, propofol pretreatment, but not post-treatment, has been shown to prevent Aqp2 downregulation and protect renal function during endotoxemia (21). n a porcine model of sepsis-induced AKI, treatment with human umbilical cord-derived mesenchymal stem cells (hUC-MSCs) resulted in a reduction in the expression of Aqp2 in the renal medulla, indicating a protective effect on renal function. Treatment with hUC-MSCs may protect against endothelial and tubular injury through the TLR4/NF-κB signalling pathway (82).

### 2.6 Aquaporins in liver injury

The liver plays many roles in sepsis and is also a target for sepsis-induced injury. A growing body of evidence from studies conducted to date indicates that the hepatic inflammatory response, oxidative stress, microcirculation coagulation dysfunction, and bacterial translocation play a pivotal role in the occurrence and development of sepsis-related liver injury (77). Septic shock and its toxins can cause hypoxic hepatitis, cholestasis due to altered bile metabolism and acute liver injury (83). In cholestasis, downregulation of AQP8 by TNF-α after LPS stimulation reduces water permeability in hepatocytes, impairing bile formation and exacerbating cholestasis (84). In addition, AQP8 can modulate hepatocellular mitochondrial function by altering water transport (85). AQP1, typically localized to portal venules, hepatic arterioles, and bile ducts in normal liver and early-stage primary biliary cirrhosis (PBC), is aberrantly overexpressed in proliferating bile ductules and arterial capillaries in advanced PBC, potentially contributing to angiogenesis, fibrosis, and the progression of portal hypertension (86). In another study, adenoviral delivery of the human AQP1 gene into rat livers improved LPS-induced cholestasis by normalising bile flow, biliary bile acid excretion and serum bile acid levels (**Figure 3E**). Although it did not alter protein expression of the canalicular bile salt export pump, hAQP1 expression enhanced its transport activity and restored canalicular cholesterol content, suggesting a potential therapeutic approach for sepsis-associated cholestatic diseases (87).

### 2.7 Sepsis induced intestinal injury

Intestinal injury occurs in sepsis, where the barrier function is frequently compromised, leading to increased permeability, bacterial and endotoxin translocation, and further intensification of the systemic inflammatory response (88). Not much is known about aquaporins in intestinal injury. In a septic mouse model induced by cecal ligation and perforation (CLP), sepsis caused intestinal injury with disrupted mucosal structure, increased intestinal ischemia–reperfusion injury, increased plasma diaminooxidase (DAO) and intestinal-type fatty acid-binding (FABP2) protein levels, and decreased AQP3 and occludin expression. Oral glycerol administration partially restored intestinal morphology, decreased intestinal ischemia–reperfusion injury, decreased DAO and FABP2 levels, upregulated occluding and AQP3 expression and improved survival compared to untreated septic mice. These findings suggest a protective role for AQP3 in sepsis-induced intestinal injury and the potential of glycerol as a surrogate for AQP3 to improve intestinal barrier function and survival (89) (**Figure 3F**).

## 3 Polymorphisms in aquaporin genes

In recent years, single-nucleotide polymorphisms (SNPs) in aquaporin genes have been linked to various pathological conditions, highlighting their significant clinical value (91–93). Notably, the -1364 A/C (rs3759129) polymorphism in the promoter region of the AQP5 gene has been extensively studied by our group in the context of sepsis (10). The initial investigation sought to ascertain the correlation between the AQP5 promoter -1364A/C polymorphism and 30-day survival in patients with severe sepsis. The findings revealed a notable increase in survival rates among patients with combined AC/CC genotypes, in comparison to those with AA genotypes. This observation remained consistent even after adjusting for various clinical covariates. The findings emphasize the potential prognostic significance of AQP5 expression variations in severe sepsis, underscoring the role of AQP5 channels in influencing patient outcomes (9). Furthermore, we demonstrated that the C-allele of the AQP5-1364A/C polymorphism, which is associated with decreased AQP5 expression and improved outcomes in sepsis, is linked to higher promoter methylation of AQP5 in neutrophils, monocytes, and lymphocytes in both septic patients and healthy controls. Furthermore, decreased AQP5 promoter methylation was correlated with increased AQP5 expression in cell-line models, indicating that AQP5 promoter methylation may serve as a crucial mechanism in genotype-dependent AQP5 expression regulation. This suggests that AQP5 promoter methylation may represent a potential target for interventions in sepsis (94). In patients with sepsis, elevated methylation levels at the cytosine site nt-937 within the AQP5 promoter are linked to augmented AQP5 mRNA expression and are predictive of an elevated risk of mortality within 30 days. This indicates that epigenetic regulation of AQP5 via NF-κB binding at nt-937 is of pivotal importance in influencing the outcome of sepsis, thereby underscoring the potential prognostic significance of AQP5 promoter methylation in septic patients (95). Aqp5 knockout (KO) mice exhibited significantly higher survival rates post-LPS injection compared to wild-type (WT) mice, indicating that Aqp5 deficiency exerts a protective effect in sepsis. Furthermore, AQP5 expression and the AQP5 -1364A/C polymorphism were observed to regulate immune cell migration, with neutrophils from individuals with the AA genotype demonstrating earlier and more precise migration compared to those with AC/CC genotypes. This suggests that AQP5 plays a role in modulating immune responses and survival outcomes in sepsis (96).

We also examined the association between complications in septic patients and the AQP5 -1364A/C polymorphism. Here we investigated the association between the promoter polymorphism and major adverse kidney events in septic patients, as well as its impact on 90-day survival. The results demonstrated that individuals with AC/CC genotypes exhibited a reduced incidence of major adverse kidney events in comparison to those with AA genotypes. Furthermore, C-allele carriers demonstrated enhanced 90-day survival rates. Subsequent multiple proportional hazard analysis substantiated the association between AC/CC genotypes and a diminished risk of mortality within 90 days, thereby corroborating the AQP5 -1364A/C polymorphism as an independent prognostic factor in sepsis, with implications for precision medicine (97). In acute respiratory distress syndrome (ARDS) caused by bacterial pneumonia, the AQP5 -1364A/C promoter polymorphism’s C-allele was linked to reduced pulmonary inflammation and improved 30-day survival rates, offering potential insights for characterizing and treating ARDS on an individualized basis. This finding highlights the impact of AQP5 genotype on inflammation and prognosis in ARDS, suggesting a significant advancement in understanding and managing this condition (98). The association between the AQP5 promoter -1364A/C polymorphism and AKI in patients with pneumonia-induced acute respiratory distress syndrome (ARDS) were examined. Results show that while the incidence of AKI upon admission did not differ between genotypes, by day 30, the AA genotype had a significantly higher prevalence of AKI compared to AC/CC genotypes. Moreover, the AA genotype was identified as an independent risk factor for AKI persistence, indicating that AQP5 genotype may influence AKI development and resolution beyond fluid balance considerations in ARDS (99).

Furthermore, a polymorphism in the AQP3 gene was examined. There was an association between AQP3 polymorphism (rs17553719) and expression with survival outcomes in sepsis patients. Results showed that the CC genotype was linked to decreased 30-day survival, higher AQP3 mRNA expression, and elevated IL-33 concentration, suggesting a potential role of AQP3 in sepsis prognosis (100). Polymorphisms in AQP genes could therefore influence the disease progression in sepsis.

## 4 Aquaporins in inflammasome activation

The role of AQPs in inflammasome activation has been described intensively in other reviews (21, 29). The inflammasome, crucial in the immune response, is found in macrophages and neutrophil granulocytes and recognizes various pathogen antigens. The NLRP3 inflammasome, upregulated in sepsis, triggers the release of IL-1β, dependent on cell pH and facilitated by aquaporin-mediated water influx in macrophages (101). The movement of water by AQPs appears to be a pivotal factor in unifying the activators of the NLRP3 inflammasome. The absence of AQP1 in a mouse model of acute lung injury resulted in a reduction in IL-1β release and neutrophilic inflammation, which serves to underscore the role of AQPs as a danger signal for NLRP3 activation. AQP3, which is highly expressed in THP-1 cells, plays a role in the rapid changes in cell volume and the activation of the inflammasome in response to stimuli such as reswelling, nigericin, and ATP. The increased expression of AQP3 serves to amplify these responses, while its peroxiporin activity has been observed to enhance intracellular ROS and inflammasome activation. Furthermore, AQP4 resulted in a reduction of NLRP3, caspase-1, and IL-1β proteins in the treatment group, indicating the inactivation of the inflammasome (102). Furthermore, the absence of AQP5 was observed to facilitate NLRP3 inflammasome activation via the generation of reactive oxygen species (ROS). The inhibition of ROS or the blockade of the NLRP3 inflammasome was observed to markedly diminish the extent of damage and pyroptosis in AQP5-deficient lacrimal gland epithelial cells (103).

## 5 Potential role of aquaporins in immune metabolism

**Figure 4:**
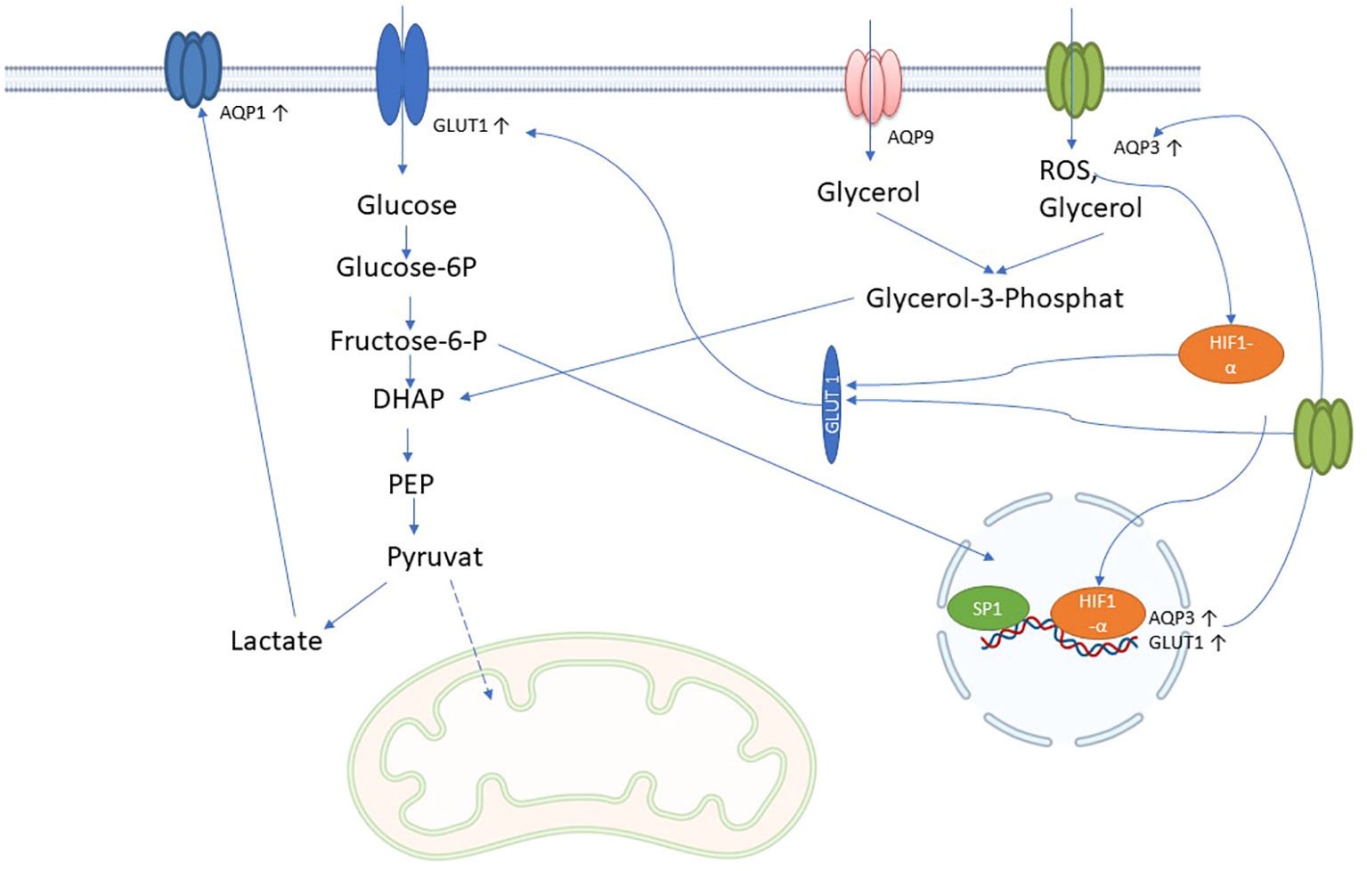
Possible role of AQPs in immune metabolism in sepsis: AQP3 and AQP9 facilitate the influx of glycerol into cells, which is converted to glycerol-3-phosphate by glycerol kinase 2 and then to dihydroxyacetonephosphate (DHAP) by glycerol-3-phosphate dehydrogenase 1. DHAP is incorporated into glycolysis and could increase glycolysis and lactate production. Lactate leads to increased expression of AQP1. In addition, AQP3 can also transport H_2_O_2_ (reactive oxygen species; ROS), which increases HIF-1 alpha expression and nuclear translocation, which in turn increases AQP3 and glucose transporter type 1 (GLUT1) expression. Increased glycolysis further increases AQP3 expression via SP1 through the hexosamine biosynthetic pathway.

The term “immunometabolism in sepsis” denotes the intricate interplay between the immune system and the body’s metabolic processes during the course of sepsis. Immune cells undergo metabolic alterations during sepsis in order to rapidly provide energy and materials for defense responses. These involve increased glycolysis and changes in fatty acid and amino acid utilization, which occur during the proinflammatory phase of sepsis (104). Similar to tubular epithelial cells (TECs), immune cells in sepsis may undergo a profound metabolic transformation, transitioning from oxidative phosphorylation (OXPHOS) to a predominance of aerobic glycolysis. Within this metabolic realignment, the majority of pyruvate generated through glycolysis avoids mitochondrial entry and is instead converted into lactate—a process catalyzed by lactate dehydrogenase (LDH). This strategic metabolic adaptation is crucial, as it supports increased ATP production through glycolysis to meet the elevated energy demands imposed by the septic challenge (105). Aquaporins (AQPs), particularly those involved in glycerol transport, play a crucial role in enhancing glycolysis during sepsis by ensuring the availability of key substrates and supporting the increased metabolic demands of cells. Specifically, aquaglyceroporins such as AQP3, AQP7, AQP9, and AQP10 facilitate the transport of glycerol across cell membranes (**Table 1**). In sepsis, the body’s need for energy surges, leading to the upregulation of these AQPs. Especially AQP3 and AQP1 seem to be upregulated in immune cells after inflammatory stimulus (106). It is known that AQP3 and AQP9 can influence gluconeogenesis by transporting glycerol into the cell (107). The glycerol transported into cells is converted into glycerol-3-phosphate (G3P) by glycerol kinase, and then into dihydroxyacetone phosphate (DHAP), an intermediate in the glycolytic pathway (108) (**Figure 4**). This process directly feeds glycerol into glycolysis, enhancing its flux and thereby increasing ATP production.

Furthermore, during sepsis, hypoxia-inducible factor 1-alpha (HIF-1α) triggers the upregulation of glucose transporter 1 (GLUT1), enhancing glucose uptake into cells (104). AQP3 supports this process by facilitating the transport of glycerol, which complements glucose metabolism (**Figure 4**). Additionally, elevated levels of O-GlcNAcylation, a post-translational modification of proteins that occurs during sepsis, further increase glucose uptake via GLUT1 and regulate glycerol transport through AQP3. This dual availability of glucose and glycerol ensures rapid glycolysis, meeting the high energy demands of immune cells (106, 109).

AQP7 also plays a significant role in metabolic regulation by influencing lipid metabolism and glycerol availability. In sepsis, increased intracellular glycerol levels or active AQP7 expression could enhance p38 signaling, which is associated with the upregulation of glycolysis and nitric oxide production. This metabolic flexibility might allow immune cells to adapt to the energy demands of the septic environment (110).

The interaction between glycolysis and glycerol metabolism is crucial in sepsis. For instance, glycerol processed through glycolysis can enter the pentose phosphate pathway (PPP), which is important for producing NADPH and ribose-5-phosphate, essential for biosynthesis and redox balance in immune cells. AQP1, although primarily known for water transport, is upregulated by glycolysis (**Figure 4**), may also indirectly influence glycolysis by regulating glucose availability and interacting with other metabolic pathways, including those involving lactate and hydrogen ion (H+) transport, which are byproducts of glycolysis (111).

In summary, aquaporins contribute to the increased glycolysis observed in sepsis by facilitating glycerol transport, supporting glucose uptake, and interacting with various metabolic signaling pathways. This enhancement of glycolysis ensures that immune cells have sufficient energy to respond to infection and maintain cellular function under the stress of sepsis.

## 6 Aquaporins as potential drug targets in sepsis

Aquaporins may be useful drug targets in sepsis. For example, we recently showed that methazolamide and furosemide reduced AQP5 expression in REH cells, with methazolamide also reducing immune cell migration. However, only methazolamide pre-treatment showed potential to reduce LPS-induced AQP5 expression, suggesting it may be useful in sepsis prophylaxis (112). AQP9, found in hepatocytes and leukocytes, is being investigated as a potential target to reduce mortality in septic shock. Aqp9 knockout (KO) mice showed prolonged survival and reduced inflammation compared to wild-type (WT) mice after LPS-induced endotoxic shock. KO mice exhibited reduced production of pro-inflammatory nitric oxide (NO) and superoxide anion (O2-), as well as reduced levels of iNOS and COX-2, which was attributed to impaired NF-κB p65 activation in various tissues. Blocking AQP9 with HTS13286 in FaO cells also prevented LPS-induced inflammation, suggesting a role for AQP9 in early phases of endotoxic shock via modulation of NF-κB signalling. These findings highlight AQP9 as a promising target for the development of new therapies against endotoxemia (113). In a study, the novel AQP9 inhibitor RG100204 was shown to normalise oxidative stress and improve survival in mouse models of sepsis. RG100204 reduced cardiac and renal dysfunction, decreased activation of the NLRP3 inflammasome pathway and reduced myeloperoxidase activity in lung tissue, suggesting that AQP9 is a potential drug target for polymicrobial sepsis (55). Another study investigated the role of FGD5-AS1 in sepsis and LPS-induced inflammation and showed that FGD5-AS1 overexpression increased AQP1 and decreased miR-133a-3p expression, subsequently reducing inflammatory cytokines such as TNF-α, IL-6 and IL-1β. Dual-luciferase reporter and miRNA pull-down assays confirmed that FGD5-AS1 acts as a competitive endogenous RNA for miR-133a-3p on AQP1, suggesting that overexpression of FGD5-AS1 may inhibit the inflammatory response in sepsis (114). Another study investigated the relationship between AQP1, miRNA-874 and lncRNA H19 in LPS-induced sepsis. It was found that H19 and AQP1 expressions decreased while miR-874 expression increased in sepsis samples, mouse models and cardiomyocytes. H19 acted as a competitive endogenous RNA (ceRNA) for AQP1 by regulating miR-874, reversing LPS-induced inflammatory responses and myocardial dysfunction, suggesting H19 as a potential therapeutic target for sepsis-associated myocardial dysfunction (54). The development of aquaporin (AQP) inhibitors faces significant challenges, particularly due to potential off-target and side effects. Many putative AQP modulators reported in literature have failed to show consistent activity upon retesting (115). This inconsistency is often attributed to the limitations of assays used, such as oocyte swelling or calcein fluorescence, which are prone to artifacts (116). Apparent inhibition of osmotic swelling may result from factors unrelated to AQPs, such as changes in cell volume regulation or the activity of non-AQP ion or solute transporters. Common inhibitors of ion transport processes, like bumetanide or acetazolamide, may alter resting cell volume, further complicating the assessment of true AQP inhibition (117). The complexity of identifying specific AQP inhibitors is compounded by the structural characteristics of AQPs and their high abundance in cell membranes. In some cases, reported inhibitors, such as loop diuretics or antiepileptics, have confused the literature due to their lack of specificity and inability to be confirmed in subsequent studies (115). For example, AER-270, a claimed selective AQP4 inhibitor, showed only partial inhibition in mouse and human models, and its effects may be due to its role as an NF-κB inhibitor rather than directly targeting AQP4 (118, 119). This highlights the need to thoroughly evaluate off-target effects, as inhibitors may influence pathways beyond AQPs. This underscores the critical need to better understand these unintended interactions to develop more selective and effective AQP inhibitors in the future (120). Further development of AQP-targeted therapeutics requires well-designed, large-scale functional screens to identify true small-molecule inhibitors. Additionally, targeting AQP signaling pathways or intracellular trafficking, as seen with vasopressin receptor antagonists like vaptans, may offer alternative therapeutic approaches. Nonetheless, off-target effects remain a significant concern, underscoring the need for careful validation in future studies (17, 116). It is possible that another potential drug under investigation, phloretin, may be able to interfere with AQPs. *In vitro* studies have shown that inhibition of AQP9 with phloretin can reduce mortality, inflammatory responses and organ damage in sepsis models (38).

## 7 Conclusion

AQPs are emerging as crucial players in sepsis, influencing various organ systems. Their roles in immune cell activation, fluid regulation, and inflammatory processes make them attractive therapeutic targets. In sepsis, AQP1, AQP4, AQP5, and AQP9 have been shown to significantly affect organs like the brain, heart, lungs, kidneys, and liver. For example, AQP4 plays a key role in SAE by contributing to cerebral edema, while AQP1 and AQP9 are implicated in myocardial injury and ALI. Modulating these AQPs shows potential to alleviate organ damage and improve patient outcomes.

Therapeutic strategies have been developed to target these proteins. Dexamethasone and traditional Chinese medicines have shown potential in reducing cardiac and pulmonary damage. Specific inhibitors such as HTS13286 and RG100204 targeting AQP9 have demonstrated promise in reducing inflammation, improving survival, and mitigating organ dysfunction in sepsis models. These approaches highlight the therapeutic potential of modulating AQPs in sepsis-related complications like ALI, SAE, and AKI, which affects around 50% of sepsis patients.

However, the development of AQP inhibitors faces significant challenges due to potential off-target effects. Many inhibitors reported in the literature have shown inconsistent activity, often complicated by nonspecific interactions with other ion transporters or signaling pathways. Future research must focus on refining these inhibitors, exploring alternative pathways such as intracellular trafficking, and conducting large-scale screening to discover more selective and effective therapeutic options.

In summary, AQPs represent promising biomarkers and therapeutic targets in sepsis, especially in modulating inflammation and organ injury in critical systems such as the brain, heart, lungs, kidneys, and liver. However, the complexity of their inhibition and the risk of off-target effects necessitate further investigation into selective therapeutic approaches.
